# Molecular Targets of Aspirin and Prevention of Preeclampsia and Their Potential Association with Circulating Extracellular Vesicles during Pregnancy

**DOI:** 10.3390/ijms20184370

**Published:** 2019-09-05

**Authors:** Suchismita Dutta, Sathish Kumar, Jon Hyett, Carlos Salomon

**Affiliations:** 1 Exosome Biology Laboratory, Centre for Clinical Diagnostics, University of Queensland Centre for Clinical Research, Royal Brisbane and Women’s Hospital, The University of Queensland, Brisbane, QLD 4029, Australia; 2 Departments of Comparative Biosciences and Obstetrics and Gynecology, University of Wisconsin, Madison, WI 53792, USA; 3 Royal Prince Alfred Hospital Sydney, University of Sydney, Camperdown, NSW 2050, Australia; 4 Maternal-Fetal Medicine, Department of Obstetrics and Gynecology, Ochsner Clinic Foundation, New Orleans, LA 70124, USA; 5 Department of Clinical Biochemistry and Immunology, Faculty of Pharmacy, University of Concepción, Concepción, Region Bio-Bio 4070386, Chile

**Keywords:** pregnancy, placentation, preeclampsia, low dose aspirin, exosomes

## Abstract

Uncomplicated healthy pregnancy is the outcome of successful fertilization, implantation of embryos, trophoblast development and adequate placentation. Any deviation in these cascades of events may lead to complicated pregnancies such as preeclampsia (PE). The current incidence of PE is 2–8% in all pregnancies worldwide, leading to high maternal as well as perinatal mortality and morbidity rates. A number of randomized controlled clinical trials observed the association between low dose aspirin (LDA) treatment in early gestational age and significant reduction of early onset of PE in high-risk pregnant women. However, a substantial knowledge gap exists in identifying the particular mechanism of action of aspirin on placental function. It is already established that the placental-derived exosomes (PdE) are present in the maternal circulation from 6 weeks of gestation, and exosomes contain bioactive molecules such as proteins, lipids and RNA that are a “fingerprint” of their originating cells. Interestingly, levels of exosomes are higher in PE compared to normal pregnancies, and changes in the level of PdE during the first trimester may be used to classify women at risk for developing PE. The aim of this review is to discuss the mechanisms of action of LDA on placental and maternal physiological systems including the role of PdE in these phenomena. This review article will contribute to the in-depth understanding of LDA-induced PE prevention.

## 1. Introduction

Pregnancy is an important event that leads to significant changes in maternal physiology. Successful pregnancy requires involvement of a series of processes commencing from fertilization to establishment of placental and maternal vascular connection with the fetus in correct order. Adequate placentation is one of the prerequisites for maintaining a normal healthy pregnancy. New insights into the placentation process involve migration, invasion, adherence, proliferation and differentiation of the placental principal cellular component, i.e., extravillous trophoblasts (EVTs), followed by their interaction with the pre-decasualized maternal uterine blood vessels, glands and lymphatics [1]. Placentation further evolves by digestion of the extracellular matrix where the EVTs tolerate surrounding maternal circulatory oxidative stress and the effects of soluble cytokines [1]. Nonetheless, the allogenic EVTs also interact with maternal decidual immune cells to provide immune competence [2]. Any deviation in these events may lead to pathological pregnancies, i.e., preeclampsia (PE). The broad concept of PE pathophysiology includes defective trophoblast invasion and inadequate uterine spiral arterial remodeling in the first trimester that follows with reduced uteroplacental perfusion [3]. This subsequently leads to poorly perfused and stressed placental syncytiotrophoblasts that release a range of mediators causing endothelial dysfunction and PE clinical manifestations [3]. Moreover, such abnormal placentation leads to the secretion of abnormal levels of anti-angiogenic and inflammatory proteins that enter the systemic maternal circulation and impair maternal systemic vascular function, resulting in the clinical manifestations of PE. Since PE and its clinical symptoms rapidly abate after delivery (removal of the placenta), the placenta must play a central or initiating role in this pregnancy disorder. 

Novel pharmacological interventions for the prevention of PE have not been developed for many years, as the complex pathophysiology, diversified clinical presentation of the disease and difficulties associated with conducting drug discovery research in pregnant women have hampered their development. Low dose aspirin (LDA) is considered to be the most effective prophylactic therapy for reducing disease prevalence in women at high risk for developing early-onset PE. The use of LDA in pregnant women is generally considered to be safe as it does not affect the pregnant mothers and/or their unborn fetuses inadvertently. It has been suggested that the principal mechanism of action by which LDA exerts its effect is via the inhibition of thromboxane production that leads to the inhibition of platelet aggregation. Additionally, LDA has a direct positive effect on the villous trophoblasts [4]. However, recent evidence suggests that LDA prevents the development of PE by promoting trophoblast invasion and migration into the uterine arteries, interfering with cytokine production and stimulating the production of proangiogenic protein placental growth factor (PlGF); thereby, inhibiting apoptosis and premature uterine arterial remodeling [5]. 

Recent meta-analysis suggest that LDA (≥100 mg/day) in early gestation (before 16 weeks) is beneficial in preventing common pregnancy complications; i.e., PE, fetal growth restriction, preterm birth [6,7,8,9], suggesting that aspirin may have effect on implantation and early placentation [10]. Low-dose aspirin has been utilized for many years to prevent PE [11,12,13]. A recent individual patient data meta-analysis observed that LDA can reduce the risk of PE development by 10% and small for gestational age (SGA) births by 24% [14] without posing a major safety risk to mothers or fetuses other than placental abruption in some cases [15]. Other studies reported that low dose aspirin is generally well tolerated within both preconception and early pregnancy periods [16]. 

**Figure 1:**
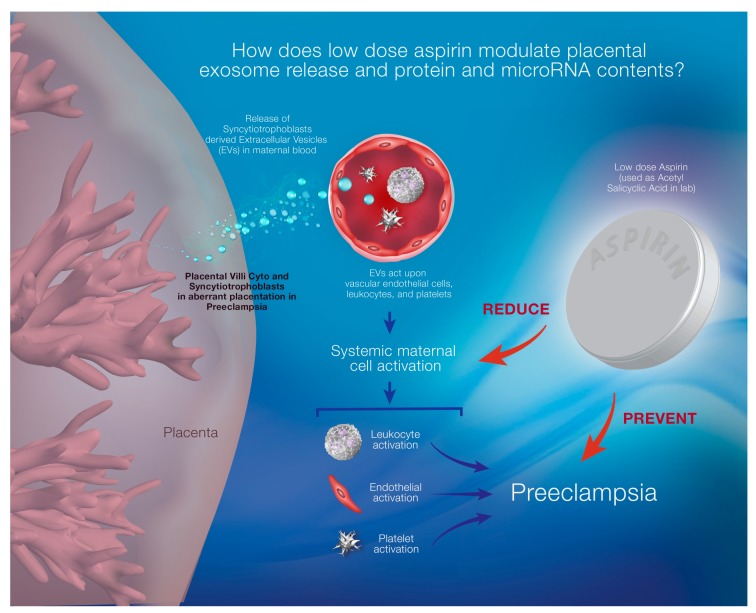
Diagrammatic representation of preeclampsia (PE) development pathogenesis and mechanism of prevention by low dose aspirin (LDA). In PE, syncytiotrophoblast-derived extracellular vesicles (EVs), including exosomes, are released into the maternal circulation in increased amounts due to inadequate placental vascular remodeling. These EVs activate the vascular endothelial cells, leukocytes and platelets and cause dysfunction. LDA prevents the development of PE by reducingendothelial cell dysfunction. The proposed mechanism that was investigated; acetylsalicylic acid, the crude form of aspirin, modulates trophoblast derived exosome release and changes their proteomic and microRNA contents.

In normal healthy pregnancy, placental syncytiotrophoblast release extracellular vesicles (EVs) including exosomes into the maternal bloodstream that contain some information (i.e., micro RNA, mRNA, proteins) to convey from the originating cells to their distant target cells such as maternal immune cells in order to adapt to the pregnancy associated physiological changes [17]. This EV release is further increased from the preeclamptic placenta due to oxidative stress, causing widespread systemic endothelial dysfunction, giving rise to maternal hypertension, feto-placental circulatory compromise and damaging various maternal organs [17]. Some recently published reports have suggested that LDA could influence platelet derived EV release; however, the effect of LDA on the regulation of placental EV release is not known. Therefore, in this review, we will discuss the potential mechanisms of action of aspirin in the context of PE prevention and the potential role of extracellular vesicles released from the placenta in this phenomenon (Figure 1).

## 2. Physiology of Pregnancy

Pregnancy induces a number of alterations to maternal physiology for maintaining the correct course of pregnancy and it involves a cascade of processes commencing from fertilization to the establishment of feto-maternal communication and cross-talk mediated via the placenta. Preimplantation conditions, vascularisation, invasion of the embryonic cells to the maternal uterine wall and oxidative stress are the essential regulators in the function of pregnancy events [18]. Prior to implantation, there is a postovulatory surge of circulatory progesterone level that inhibits the proliferation of estrogen-dependent uterine epithelium and induces secretory transformation of uterine glands. In the early stages of development, i.e., ~day 6 of fertilization, the microvilli of the blastocyst interact with the pinopodes of the uterine endometrial luminal epithelium in order to establish apposition, which becomes stable through the increased adherence of the trophectoderm and the uterine luminal epithelium. During this interaction, a range of molecules are secreted from the immune activated cells including mucin, selectin, integrin and cadherin [18,19]. Shortly thereafter, invasion begins and trophectoderm penetrates the uterine epithelium, invading the wall of the uterine arteries where they interact with the cells of the maternal circulatory immune system and mediate the remodeling of the uterine spiral arteries that supply the placenta. This is followed by deportation of aggregates containing transcriptive materials.

There is direct evidence that platelets are involved in the placentation process. During placentation, trophoblasts invade the decidual stroma including the uterine glands and migrate into the maternal uterine spiral arteries replacing the vascular smooth muscle cells to remodel the arteries as low resistance, large caliber vessels [20]. This process ensures adequate placental perfusion by the remodeling of maternal uterine vessels. Histological examination shows deposition of maternal platelets in the trophoblast aggregates formed in the uterine spiral arteries. A number of studies discovered that these platelets are activated, releasing soluble factors enhancing the invasive capacity of trophoblast cells [21]. The trophoblasts produce a range of vasoconstrictors and vasodialators that are in balance to maintain the placental blood flow for proper fetal development during pregnancy [22].

During pregnancy, the viscosity and coagulability of maternal blood upsurges due to the increase in pro-coagulant agents, such as plasminogen activator inhibitor-1, fibrinogen, factor VII, VIII, von Willebrand factor and to the reduced fibrinolysis. The hypercoagulable state is attributed to the activation of platelets [23]. Maternal serum biochemical markers of pregnancy, namely alpha fetoprotein (AFP), human chorionic gonadotrophin (hCG), unconjugated estriol [24] and inhibin-A [25], are produced and found in higher concentration in the maternal peripheral circulation due to the implantation and placentation processes of pregnancy.

## 3. Pathogenesis of Preeclampsia

Preeclampsia (PE) is defined as new onset of hypertension after 20 weeks’ gestation with renal, hepatic, hematologic, neurological, pulmonary or fetal involvement. Physical signs of preeclampsia are hypertension, proteinuria, renal insufficiency, hemolysis, reduced platelet count and/or increased platelet activation [26]. It is a serious complication of pregnancy affecting ~7.6% pregnancies globally and is associated with high morbidity and mortality in affected mothers and children [27]. It is a lifelong disorder with increased risks of neonatal and child morbidity and mortality including health risks in adulthood [27]. Pregnancy induced hypertension is one of the most prevalent risk factors for the development of PE. The consequences of PE include intrauterine fetal growth restriction (IUGR) and preterm birth. [28].

In PE, there is widespread systemic endothelial dysfunction that leads to hypertension and concomitant proteinuria [29]. Clinical risk factors for developing PE assessed before 16 weeks of gestation include prior history of hypertension, chronic hypertension, pre-gestational diabetes, pre-pregnancy BMI > 30 and use of assisted reproductive technology [30]. The most commonly used screening test for early prediction of PE involves analysis of maternal characteristics, maternal mean arterial pressure, uterine arterial Doppler pulsatility index and serum biochemistry (PaPP-A and/or PlGF). This test is performed at 11–13 weeks of gestation [31]. The present management of patients with PE depends on symptom severity. Currently, some drugs are available to treat mild to severe PE (e.g., methyldopa, hydralazine, magnesium sulphate) [32]. However, the best treatment currently available for PE is delivery of the newborn and placenta as all the signs and symptoms of PE abolish when the placenta is separated from the mother. 

In early-onset PE, there is defective implantation and placentation due to inadequate extravillous trophoblast invasion and partial failure of uterine arterial remodeling, resulting in high resistance and low capacitance vascular supply to the placenta and fetus [33]; however, this phenomenon is not evident in late-onset PE. Some research studies showed that during normal healthy pregnancy, the invasive trophoblast cells replace the smooth muscle and elastic lamina of the maternal uterine vessels, causing dilation and funneling at the vessel mouth and facilitating further migration of trophoblasts. In the absence of conversion of the maternal uterine vessels, there is retention of smooth muscle cells contributing to increased resistance to maternal blood flow. Nonetheless, maternal blood enters into the intervillous space as a turbulent jet that increases the risk of spontaneous vasoconstriction and ischemia-reperfusion injury, generating oxidative stress within the maternal circulation [34]. This in turn gives rise to placental villous infarcts, constriction of spiral arteries due to the mural hypertrophy and fibrin deposition, leading to the abnormal ultrasound indices and biochemical markers seen in the maternal circulation. This failure in vascular dilation has a direct impact on placental blood flow and is the primary determinant of pregnancy pathology [34]. In addition to the general concept of PE pathogenesis, where there is defective EVT invasion and uterine arterial remodeling, there is also an imbalance of angiogenic and antiangiogenic factors. These factors include vascular endothelial growth factor (VEGF), soluble endoglin, soluble fms-like tyrosine kinase-1 receptors (sFlt-1) and placental growth factor (PlGF). Abnormal production of these factors is closely associated with PE and intrauterine growth restriction [35]. At the end of the first trimester of pregnancy, the extravillous trophoblasts (EVT) invade the uterine spiral arteries and replace the vascular smooth muscle and the endothelium to remodel the arteries, which lead to the formation of low resistance and high capacitance vessels that facilitates increased placental perfusion. When there is perturbation of this process, there is reduced placental perfusion causing placental stress where platelets aggregate and accumulate in the partially damaged placenta [36]. In PE, there are interactions between maternal characteristics and risk factors and placental pathophysiological factors leading to a vicious cycle of maternal inflammation, vascular dysfunction and the activation of pro-coagulation pathways [33].

Current research on PE is focused on the role of extracellular vesicles released from the placenta [33]. Following placentation, the residual syncytiotrophoblastic material generated from placental shedding or by placental microparticles releases various vessel constricting factors that cause systemic endothelial dysfunction [37,38]. These microparticles contain a set of proteins including some pro-inflammatory and pro-coagulatory molecules that contribute to the development of PE [39,40,41] A recent article on PE stated that there was interaction between fetal Human Leukocyte Antigen-C (HLA-C) molecule and maternal natural killer cells’ killer-cell immunoglobulin-like receptor (KIR) in severe PE; these molecules are carried by the EVs released from the placenta and maternal circulatory cells [42]. Inadequate placentation causes the development of pregnancy-induced hypertension (PIH) and preeclampsia (PE) [43,44], leading to focal regions of hypoxia that are responsible for modifying the production of growth factors, cytokines [45], lipid peroxides [46] and prostaglandins by placental trophoblasts [45]. Elevated placental levels of inflammatory cytokines, such as tumor necrosis factor-α, interleukin (IL)-1α, IL-1β and IL-6, are generally considered unfavorable to pregnancy [47]. Moreover, clinical studies have shown changes in the levels of cytokines and prostaglandins in women with PE [48,49]. Maternal circulatory neutrophils are activated in pregnancy and further activated in PE, which are the source of oxidative stress by generating reactive oxygen species such as hydrogen peroxide and superoxide anion and these molecules cause damage to the proteins, lipids and nucleic acids [50]. Neutrophil activation is initiated in the intervillous space by increased secretion of lipid peroxides by the placenta, which is abnormally increased in PE. This stimulates phospholipase A2 and cyclooxygenase enzymes to increase the production of thromboxane. Thromboxane is implicated in monocyte activation responses and plays role in mediating tumor necrosis factor alpha (TNF-α) production by neutrophils in response to oxidative stress [50].

In PE, the production of thromboxane A2 and prostaglandin I2 is altered with excessive accumulation of THXA2 metabolite in the maternal systemic circulation [51,52]. This results to the increased activation and aggregation of platelets and vasoconstriction causing impaired placental perfusion and oxidative stress [53,54,55,56,57]. The platelet count is reduced in PE due to platelet activation and aggregation under the effect of elevated levels of ThXA2 Synthase [28]. In addition [36], PE contributes to some biochemical changes in maternal circulatory system, such as, increase in phosphodiesterase-5 [58], thromboxane synthase [28] and an elevated hCG level [59]. Nonetheless, immunological changes also take place in PE. There is rise in anti β_2_-glycoprotein I antibodies that are related with aberrant implantation [60]. many predictive biomarkers for PE have been described including placental biomarkers (PAPP-A, PLGF, s-FLT-1, placental protein 13 (PP 13)), Free HbF, Alpha 1 Macroglobulin and Uterine Artery Doppler Pulsatility Index [61,62]. Not all studies have consistently shown value of these markers, for example changes in PP13 have not been consistently replicated [63]. Measurements of total cell free DNA and fetal fraction in maternal plasma at 11–13 and 20–24 weeks are not predictive of PE [64].

## 4. Pharmacology of Aspirin and Basis for Its Use in PE

The chemical name of aspirin is acetylsalicylic acid (ASA) [65,66,67,68,69]. It is a nonsteroidal anti-inflammatory drug (NSAID). It is typically used in two dose regimens—high dose (600 mg) and low dose (60–150 mg). It has anti-inflammatory, analgesic, antipyretic and antiplatelet effects [70]. The endothelial dysfunction in PE involves increased lipid peroxidation, which activates COX and inhibits prostacyclin synthase, thus inducing rapid imbalance in the TXA2/prostacyclin (PGI2) ratio in favor of TXA2 [51]. TXA2 favors systemic vasoconstriction, and increasing platelet aggregation and adhesion, which is compensated in this context by the vasodilator effect of prostacyclins, levels of which drop sharply. This imbalance is present from 13 weeks of gestation in high-risk PE patients [71]. LDA treatment for 2 weeks reverses TXA2/PGI2 imbalance by inhibiting THXA2 production [72,73]. Some studies observed that LDA can reduce the release of sFLT-1 from trophoblast cells and induce the production of vascular endothelial growth factor thereby promoting angiogenesis [74]. LDA also modulates cytokine production, reduces apoptosis and alters cell aggregation and fusion thereby improving defective trophoblast implantation [5]. LDA improves EVT migration and invasion into the maternal uterine spiral arteries and reduces placental cell apoptosis [75]. PE is associated with some augmented anti-angiogenic, oxidative and pro-inflammatory markers, as well as increasing human polymorphonuclear neutrophil (PMN)-endothelial cell adhesion [76]. LDA reduces the circulatory levels of these factors and improves the cytokine profile [5]. LDA causes retardation in leukocyte-endothelial cell adhesion and interaction and thus it prevents the endothelial cell dysfunction in PE [76]. Several reports have suggested that few biomarkers can be identified in maternal blood to be monitored for assessing treatment response after initiation of LDA treatment in pregnant women at high risk for preeclampsia; i.e., placental growth factor, placental protein 13, alpha fetoprotein [77]. 

The mechanism of action of aspirin involves a cascade of events. Aspirin irreversibly acetylates the platelet enzyme cyclooxygenase (COX), modifying the production of different prostaglandins and also acts as an analgesic, anti-inflammatory agent. There are three isoforms of COX enzyme upon which aspirin acts; the sources of these enzymes are mainly platelets, but they are also found in other immune cells namely leukocytes, monocytes and macrophages. Aspirin inhibits COX-1 irreversibly and COX-2 reversibly to a lesser extent. The resultant inhibition of COX-dependent generation of thromboxane A2 prevents platelet aggregation. This effect is maintained for the entire platelet lifespan of 8–9 days [78].

## 5. Low Dose Aspirin (LDA) and Pregnancy

Low dose aspirin reduces the mortality and morbidity in pregnant women at high risk for PE [79,80,81,82]. National guidelines typically suggest that women considered to be at high risk of developing pre-eclampsia should be treated with prophylactic low dose aspirin to reduce the prevalence of disease, although there are differences in how “high risk” is defined (NICE guidelines and ACOG recommendations (2017)). Acetylsalicylic Acid (ASA) is considered a highly attractive pharmacological agent to use in pregnancy for the prevention of maternal and perinatal mortality and morbidity worldwide due to its low cost, widespread availability, ease of administration and safety profile [83]. Aspirin is listed as a US Food and Drug Administration (FDA) category C drug during the first and second trimester and a category D drug in the third trimester of pregnancy [70]. Although some recent evidence has suggested that aspirin can affect the fetus adversely causing congenital anomaly, the FDA has assigned this drug as pregnancy category C, and treatment is relatively safe [84]. Although aspirin can cross the placenta, it is safe in low doses [85]. 

Low dose aspirin is a very effective treatment. Meta-analysis of a series of >30 randomized controlled trials have shown that low dose aspirin prophylaxis (any dose, any gestation) reduces the incidence of PE by 10% [11,12,14,86]. If analysis is restricted to assessment of outcomes for PE leading to delivery before 34 weeks in women who commence aspirin <16 weeks gestation and have a higher dose (>100 mg/day), then the data show a 90% reduction in early PE [87]. Aspirin also appears to be effective at reducing the prevalence of intrauterine growth restriction (IUGR); once again, this meta-analysis shows that treatment is more effective if a higher dose (>100 mg/day) is given and treatment is started before 16 weeks [88]. Other meta-analyses have also shown that low dose aspirin may be effective in preventing spontaneous preterm birth [6,7,8,9]. Other studies have demonstrated that low dose aspirin is generally well tolerated in both preconception and early pregnancy periods [16].

To date, several studies have attempted to assess the beneficial effects of aspirin treatment in gestational hypertensive disorders, in particular PE. In spite of different conflicting results on the effects of aspirin in pregnancy, one study found that aspirin administered early i.e., from the eighth week of gestation has in fact a positive effect on the pregnancy outcome without the manifestation of teratogenicity or fetotoxicity [29]. Recent studies on PE found that in high risk pregnancies, any preventative treatment should be aimed at or before 16 gestational weeks to be effective as placentation and uterine spiral arterial remodeling is completed by 20 gestational weeks [11]. Additionally, to prevent perinatal death and to improve perinatal outcomes, low dose aspirin should be prescribed before 16 gestational weeks [89]. Cost benefit analysis in a US based research study showed that aspirin prophylaxis through pregnancy would reduce morbidity and mortality, leading to a reduction in health care costs [90]. 

Following the preparation of a systematic review, the US Preventive Service Task Force recommended the use of low-dose aspirin (81 mg/d) as preventive medication after 12 weeks of gestation in women who are at high risk for PE [91,92,93]. The US Preventive Service Task Force also found that LDA prophylaxis in early pregnancy does not increase the chances of placental abruption, postpartum hemorrhage, fetal intracranial hemorrhage or perinatal mortality [94]. 

Other authors have suggested that the dose and timing of aspirin prophylaxis is also important. Ayala et al., 2013, identified that (i) 100 mg/d ASA should be the recommended minimum dose for prevention of complications in pregnancy; (ii) ingestion of low-dose ASA should be started at ≤16 weeks of gestation and (iii) low-dose ASA should be ingested at bedtime, not during the morning. Aspirin prescribed in this way significantly regulates ambulatory blood pressure (BP) and reduces the incidence of PE, gestational hypertension, preterm delivery and intrauterine growth restriction (IUGR) [95]. 

Other agents have been used for prophylaxis against PE in high risk women, either alone or in combination with LDA. There is a significant body of literature investigating whether low molecular weight (LMWH) or unfractionated heparin can reduce rates of PE, preterm birth, perinatal mortality and small for gestational age babies when prescribed to high risk women [96,97]. Heparin is safe from a fetal perspective and does not cross the placental barrier due to its high molecular weight [85,98]. While some observational studies that have combined the use of aspirin and LMWH show significant reduction in rates of PE in very high risk groups [99,100], an individual patient meta-analysis did not show significant benefit to this intervention [101]. Calcium (1 g/day) has also been widely investigated and appears to be particularly useful in low and middle income settings where dietary calcium intake is poor [102]. Vitamin C, D, E [103,104,105], fish oil/omega 3, statins [35], L-arginine [106] and antihypertensive drugs such as calcium channel blockers [107] have also been investigated, although there is a paucity of randomized controlled trial-based data for these investigations. The most significant ongoing research issues are to establish why aspirin is less effective in some groups of women; for example, those that have chronic hypertension and to determine whether additional agents can impact rates of term pre-eclampsia, which are not as significantly reduced using aspirin therapy. 

A table on recent studies involving aspirin and pregnancy has been presented in the Table 1.

## 6. Effects of LDA on Placental and Maternal Body System Function

To date, a number of studies have attempted to elucidate the role of aspirin in the prevention of adverse pregnancy outcomes. However, the particular function of LDA in preventing PE and other pregnancy-induced hypertension is not clearly understood. Some in-vitro studies found that there is no specific effect of LDA or LMWH on BeWo choriocarcinoma cells when treated with forskolin except cell fusion due to the placental protein level 13 increase [122]. Some studies reported that thromboxane has been found to be involved with vasoconstriction leading to placental ischemia, thrombosis and platelet aggregation [123]. Other research studies reported that aspirin can negatively act on COX2 enzyme, thereby inhibiting thromboxane A2 production from arachidonic acid [124,125]. Interestingly, there are also data suggesting that aspirin can reduce the release of thromboxane from the trophoblasts [22].

Low-dose aspirin, which selectively inhibits TXA2 production, is used to prevent high-risk PE [28]. Low-dose aspirin, a common antiplatelet agent, usually restores prostacyclin and thromboxane levels that prevent vasoconstriction, and therefore, has been targeted as an intervention to reduce PE in at-risk women [124,126]. LDA increases the production of prostaglandin I_2_ by blocking the synthesis of thromboxane A2 [73]. This PGI_2_ increases vasodilatation and prevent thromboxane mediated damage [127]. Some studies have shown that TXA2 analogues cause hypertension in pregnancy and TXAS depletion prevents hypertension and IUGR [128]. Urine specimens of PE women show the presence of thromboxane B2, which is the metabolite of thromboxane A2 and LDA shifts the balance between THXA2 and PGI2 favoring the production of PGI2 that increases the blood flow to the placenta [129].

In normal, healthy pregnancies, uterine spiral arterial remodeling occurs at around 8 weeks of gestation and is complete by 16–20 weeks [130]. However, in PE, placentation is inadequate and under stress due to impaired uterine spiral arterial remodeling [131]. Some randomized controlled clinical trials observed that LDA is associated with improvement in uterine arterial pulsatility index when started in the first trimester of pregnancy [132,133]. Another study observed that low dose aspirin reduces the UtA Doppler pulsatility index, indicating improved blood flow [134].

In-vitro studies found an association of LDA treated trophoblast cells and an improvement in cytokine profile that prevents trophoblast apoptosis and promotes angiogenesis by increasing the production of placental growth factor (PlGF) [75]. Another similar study by Panagodage, et al. identified a number of factors that are involved in preeclampsia prevention with low dose aspirin (LDA) treatment. The authors observed that placental growth factor is significantly decreased in preeclamptic women’s sera compared to normotensive women’s sera; LDA increases trophoblast secretion of PlGF and restores abnormal cytokine (Activated Leukocyte cell adhesion molecule ALCAM, CXCL-16 and ErbB3) production by trophoblasts in PE [5]. Soluble fms-like tyrosine kinase-1 (sFLT1) is an antiangiogenic factor and its expression is increased in preeclamptic placentas and in cytotrophoblast exposed to hypoxia. Aspirin inhibits the production of sFLT1 in CTBs and this effect is mediated by the inhibition of COX-1 [74].

Preeclampsia is associated with some augmented anti-angiogenic, oxidative and pro-inflammatory markers, as well as increasing human polymorphonuclear neutrophil (PMN)-endothelial cell adhesion. This cell adhesion is reduced when human PMN are incubated with ATL (aspirin triggered lipoxin A4) [76]. This aspirin triggered lipoxin is similar to endogenously produced lipoxins but the duration of action is prolonged [135]. ATL acts as an anti-inflammatory agent; it promotes angiogenesis and causes immunosuppression and it also blocks the generation of reactive oxygen species in the endothelial cells, inhibits chemotaxis of polymorphonuclear neutrophil and the leukocyte-endothelial interaction [136,137,138,139,140] causes nuclear factor kappa B activation [137,141] and secretion of tumor necrosis factor alpha (TNF-α) in activated T cells [142]. Additionally, ATL can increase nitric oxide synthesis where the heme oxygenase-1 enzyme is also involved [143] and this effect is responsible for resolving inflammation [143]. Heme oxygenase enzyme-1 degrades heme to generate bilirubin, carbon monoxide and iron, exerting their anti-oxidant, antiapoptotic and cytoprotective actions [144]. Additionally, another recently conducted study identified that aspirin prevents TNF-alpha-induced endothelial cell dysfunction by regulating the NF-kappa B-dependent miR-155/eNOS pathway in preeclampsia [145].

The pathophysiology of PE also involves the genetic expression of the STOX1 transcription factor by extravillous trophoblasts that modulate trophoblast proliferation [146,147]. The STOX1 gene is overexpressed in human placental extravillous trophoblasts and is associated with PE pathogenesis [147,148,149]. Founds et al. [150] showed, in transcriptomic analysis, that STOX1 is overexpressed during the first trimester of pregnancies that had a preeclamptic outcome. Other studies have performed functional assays to determine the function of the STOX1 gene; using an in-vivo mouse model, this gene was found to cause severe gestational hypertension, proteinuria, an increased circulatory level of antiangiogenic factors and histological alterations in the kidney as well as the placenta [151]. These researchers also demonstrated that low dose aspirin improved maternal PE-like symptoms [152]. LDA improves uterine perfusion and favourably affects aspects of reproduction [153]. In addition, empirical introduction of LDA during in vitro fertilization (IVF) treatment improves the quality of oocytes and embyros [154]. Low dose aspirin and heparin in combination improve the live birth rate in IVF for unexplained implantation failure [155]. Low-dose aspirin effectively improves perifollicular artery blood flow and enhances oocyte quality and clinical pregnancy rates [156].

## 7. Complications of LDA for Fetuses and Mothers

A systematic evidence review by the US Preventive Services Task Force (USPSTF) identified no adverse impact on the mother or offspring during the perinatal period following aspirin use for prevention of preeclampsia [157] including no documented adverse effect on neonatal platelets [158]. However, some studies have identified adverse effects with the antenatal and perinatal use of aspirin, albeit taken at a higher dose. Potential risks associated with aspirin therapy during the third trimester include premature closure of the ductus arteriosus and hemorrhagic complications [159], subchorionic hematoma if administered in first trimester of pregnancy [117], fetal loss [160], endocrine disturbances in the human fetal testis and interference in the testicular descent [161], childhood asthma [162] and fetal complications [110]. Some research studies observed that high doses of aspirin may affect fertility, increases the risk of miscarriages and may cause fetal cryptorchidism [163,164,165]. Additionally, LDA therapy in the late gestational age has on rare occasion been reported to cause renal injury, cardiovascular abnormality such as closure of the ductus arteriosus, necrotizing enterocolitis and intracranial hemorrhage in the fetus as well as reduced breast milk supply in the mother, likely due to the inhibition of cyclooxygenase enzyme pathways [164]. The common adverse effects of aspirin in adults are significantly associated with gastrointestinal or cerebral bleeding episodes [166]. Given the risks of aspirin therapy, it is better to reserve treatment for women deemed high-risk of deep placentation related disorders rather than to prescribe it universally.

## 8. Predictive Biomarkers for Preeclampsia Cases Treated with Low Dose Aspirin

Few biomarkers have been identified in maternal blood as candidates for monitoring treatment response after initiation of low dose aspirin treatment in pregnant women at high risk for preeclampsia:

(i) Maternal serum concentrations of placental growth factor (PlGF) level are generally low in preeclampsia.
(ii) Low-dose aspirin reduces adverse pregnancy outcome such as PE and delivery before 34 weeks of gestation in pregnant women with unexplained elevated levels of alpha-fetoprotein (AFP) [167,168].
(iii) Normotension in the first trimester is associated with reduced risk of PE [169].
(iv) In a randomized controlled clinical trial conducted by Asemi Z. et al., low dose aspirin (80 mg) was administered with calcium supplementation (500 mg) in pregnant women who were at risk for PE. The treatment was continued for nine consecutive weeks before measuring high sensitivity C-reactive protein (hs-CRP), total antioxidant capacity (TAC), total glutathione (GSH) in plasma and serum glucose and insulin level. The study showed a significant difference in serum hs-CRP level and increased levels of plasma TAC and total GSH in pregnant women at risk for preeclampsia as compared to those that took placebo (did not receive any treatment), but serum insulin levels were not affected at all [170].

## 9. Extracellular Vesicles (EVs)

Extracellular vesicles (EVs) are mediators that can modify the function of target cells by transferring proteins and genomic materials to other cells; thus, EVs have an active participation in cell-to-cell communication [171]. EVs shred from a variety of cells and have a number of important physiological as well as pathological functions as they are capable of trafficking and transfecting the genetic material from cell to cell. The biogenesis and contents of EVs predominantly depends on the originating cell type and their surrounding microenvironment [172]. Several studies using electron microscopy analysis to characterize the morphology of EVs demonstrate that are spherical with lipid bilayer membrane [173,174]. The correct classification of EVs still a manner of debate and the majority of the information in the literature classify them according to their size and the different biogenesis pathways. Typically, EVs are categorized as exosomes (~40–100 nm), microvesicles (~100–1000 nm) and apoptotic bodies (~1000–5000 nm) based on their size and origin. Microvesicles and apoptotic bodies are formed directly via budding of the plasma membrane, whereas exosomes are produced via an endocytic pathway [174]. Distinction between different EVs subgroups is difficult, due to the minimal physical and morphological differences, to the lack of specific markers, and to the fact that the same cellular source may dynamically produce different class of EVs in response to different conditions [175]. Recently, the international society of extracellular vesicles has recommended classifying the vesicles according to the their size in small EVs (<100–200 nm) and medium/large EVs (>200 nm), or density (low, middle, high, with each range will defined) or their biochemical composition (e.g., CD63^+ve^) [176]. Currently, there is no single method allowing for accurate characterization and discrimination of the different EVs classes [175]. EVs can be ordinarily isolated from different biological fluids using the differential and buoyant density centrifugation methods followed by ultrafiltration/size exclusion chromatography or flow cytometry or precipitation using polymers or antibodies to enrich the pure EVs population [177]. EVs play an important role in cell-to-cell communication and influence a variety of cellular functions, including cytokine production modulation, cell proliferation, apoptosis and metabolism, by transferring their protein, lipid or messenger RNA and micro RNA molecules [173,178]. EVs can be isolated both biological fluids (e.g., plasma/serum, urine, cerebrospinal fluid, saliva, etc.) and in vitro from cell-conditioned media. Moreover, as EVs are natural carriers of bioactive molecules, different research studies are addressing the therapeutic potential of EVs due to their specific genetic material packaging capabilities [175]. Several groups have identified EVs in maternal biofluids during normal and complication of pregnancies and the potential role of EV during pregnancy have been reviewed in details by our group previously [179,180,181,182]. 

## 10. Extracellular Vesicles/Exosomes in Normal and Preeclamptic Pregnancies

Different research studies utilized a variety of experimental models (i.e., biological fluids, primary placental trophoblasts, trophoblast cell line, placental explant, placental perfusate etc.) to isolate different subpopulations (exosomes, microvesicles) of EVs and studied their role in the context of healthy as well as in pathological pregnancies. Small and large EVs originating from the placenta have been identified in maternal plasma. Concentrations of both total and placenta-derived exosomes present in maternal circulation increase across gestation [183] and are higher in complicated pregnancies (such as those affected by PE) as compared to normal pregnancies [184,185]. Interestingly, the global miRNA profile within small vesicles such as exosomes differs between normal and PE pregnancies across gestation and it is likely that PE is not only associated with changes in the circulating levels of exosomes, but also in their miRNA content [186]. Recently, Biro et al. identified that hsa-miR-210 level increased in the circulating exosomes isolated from PE pregnancies [187]. Poor placentation is associated with hypoxia and oxidative stress, which are features of PE and affects the invasion of extravillous trophoblast (EVT) and the uterine spiral arterial remodeling. Truong et al. studied whether low oxygen tension alters exosome release and the exosomal miRNA profile from HTR-8/SVneo cell line and examined their interaction with endothelial cells [188]. HTR-8/SVneo cells are commonly used as a model for EVT cells, although they are not ideal, as they contain a heterogenous population of trophoblast and stromal cells [189]. In this study, low oxygen tension to exosomes from EVTs cultured under normoxic conditions. Moreover, a specific set of miRNAs within exosomes from EVTs cultured under hypoxia were identified, and these miRNAs are present in circulating exosomes at early gestation from women who develop PE later in pregnancy. This data suggests that aberrant extracellular vesicle signaling is one of the common factors in the development of PE. In normal healthy pregnancy, syncytiotrophoblast derived EVs release into the maternal blood stream where they act upon their target endothelial cells and circulating immune cells [33,190,191,192]. Placental EVs carry different proteins, lipids and nucleic acids that play a crucial role in feto-maternal communication to maintain pregnancy [193]. Interestingly, concentrations of large EVs gradually increase through pregnancy irrespective of their origin [186] and these EVs convey pro-inflammatory and pro-thrombotic antigens that might contribute to the hypercoagulable state observed in the last trimester of pregnancy [186]. Chang et al. identified that high levels of preeclamptic exosomes contain abundant sFlt-1 and sEng that can induce vascular dysfunction as these proteins were captured by vascular endothelial cells [194]. Tannetta et al. investigated the level of expression of placental protein 13 in syncytiotrophoblast derived extracellular vesicles (STBEVs) isolated from PE and normal pregnancy placental perfusate and found it was low in PE placenta [195]. Tong et al. described a novel mechanism by which placental EVs can attenuate PE pathogenesis in the presence of antiphospholipid antibody (aPL), which can induce the synthesis of toll-like receptors on placental EVs to increase the level of expression of mitochondrial DNA in these vesicles [196]. Thus, placenta-derived EVs are involved in gene regulation, placental homeostasis and cellular function that overall reflect the placental-maternal crosstalk [197]. Placental exosomes were also observed in fetal blood and their concentration correlated with fetal growth [198]. The concentration of placental exosomes in the fetal circulation was higher than that found in the maternal circulation and was also higher in pregnancies affected by PE [199]. Interestingly, not only the concentration of circulation exosomes in PE is different compared with normal pregnancies, and specific changes in the protein cargo of exosomes in PE have been identified [200]. 

Another recent study measured the level of different biomarkers including copeptin, annexin V and placental growth factor in maternal serum derived microparticles at 10–14 gestational weeks in women with PE and compared with that of normal healthy pregnancy [201]. Interestingly, the levels of nitric oxide synthase enzyme in the STBEVs were lower in STBEVs from PE compared to normal pregnancies [202]. In this regard, in a similar study, the levels of the protein neprilysin were increased in EVs of PE placenta [203]. 

Kohli et al. identified a novel pathway by which the placental EVs interact and causes release of EVs from endothelial cells and platelets that further activate the inflammasome in the trophoblast resulting in the development of PE [204]. The role of EVs in relation to PE pathophysiology including their different contents has been summarized in Table 2.

A number of drugs that can be used to treat PE appear to modulate EV expression. Some studies also addressed the mode of action of different antihypertensives, including thiazide diuretics that are used to treat the hypertension in PE. Hu et al. identified some changes in the sodium transporters in the renal tubule that were incorporated in the urinary exosomes isolated from PE women [206]. Another very interesting study by Chamley L. et al. identified melatonin as an effective agent that can reduce the endothelial cell activating placental EVs release in PE [209]. In a similar study, transthyretin which is the thyroxin binding protein, was found in aggregated form and packaged in the small placental EVs in PE [210]. Xu et al. identified potential molecular mechanisms by which vitamin-D can reduce oxidative-stress induced PE [213]. Among the different therapeutic agents, the efficacy of aspirin was evaluated due to its availability and cost-effectiveness. However, there is lack of understanding in the mechanism of action of aspirin in the context of EV secretion regulation.

## 11. Effects of LDA on Exosomal Secretion

Up until now, there has been very limited evidence on the potential effect of aspirin on the release and content of EVs. Goetzl E.J. et al., discovered that in the presence of some coagulation factors (e.g., thrombin/ collagen) induces changes in the plasmatic levels of platelet-derived exosomes and their protein content (i.e., α-granule chemokines CXCL4 and CXCL7 and cytoplasmic high-mobility group box 1 (HMGB1)) [214]. Incubation of normal platelets with aspirin significantly inhibits arachidonic acid (AA)-induced platelet reactivity, EV formation and pro-coagulant activity [215]. Interestingly, aspirin therapy can significantly reduce microparticle (MP) shredding from erythrocytes, monocytes and vascular smooth muscle cells, reversing the effects of diabetes-induced stress on these cells [216]. Other studies have identified that aspirin changes the miRNA profile and EV release from platelet [217]. Syncytiotropholast-derived extracellular vesicles that are placental alkaline phosphatase (PLAP) positive inhibit the aggregation of platelets that were treated with aspirin [218]. Tannetta et al. observed that STBEV that are placental alkaline phosphatase (PLAP) positive inhibit the aggregation of platelets that were treated with aspirin [218]. On the contrary, platelets were activated and thrombus formation was increased by the STBEV isolated from preeclamptic placentas. Another study observed the effect of anticoagulant therapy (treatment with either unfractionated heparin (UFH) or low molecular weight heparin (LMWH), and/or LDA) on cell derived microparticles and outcome of pregnancy [219]. These findings indicate that placenta-derived extracellular vesicles may provide understanding in their potential role in low dose aspirin induced placental functions. Although there is significant evidence for dysregulation of both concentrations and bioactivity of circulating placental EVs in PE compared to normal pregnancies, no studies have described the potential effect of aspirin on EVs released from placental cells and their bioactivity. 

## 12. Summary

Several studies focused on EVs (mainly small EVs called exosomes) highlighting their extraordinary characteristics as natural carriers of bioactive molecules, which can be used as biomarker for several pathological conditions including PE. These vesicles are unique in terms of their cell trafficking and transfecting capabilities. These nanovesicles are released from almost all types of cells into different human body fluids. Their release and contents are dependent on the microenvironment where the cells are exposed and the origin of the cells [172]. Therefore, EVs can be used as a diagnostic tool as well as prognostic marker for several pathologies, and we have proposed that the analysis of placental vesicles in maternal plasma can function as a liquid biopsy to establish placental function during pregnancy. During pregnancy, placenta and other cells, such as platelets and immune cells, secrete exosomes into the maternal circulation; this process is exaggerated in pathological pregnancies (i.e., PE, PIH, IUGR) in an attempt to modulate the pathology [43,190,191]. Very few studies [218,219] have been conducted to identify the particular mechanism of action that exosomes can produce on the placenta and overall maternal physiological system when treated with low dose aspirin and other antithrombotic medication. An avenue is open to explore the placental and other cell derived exosomal functions in placental dysfunctional disorders when treated with antithrombotic medications including low dose aspirin. A better understanding of these processes may lead to the development of novel prognostic markers utilizing placenta specific exosomes or for monitoring the response to aspirin treatment for placental pathologies.

## Figures and Tables

**Table 1:** Recent Studies on Aspirin and Pregnancy.

Study Design	Mode of Treatment	Outcome of Study	Reference
Randomized controlled trial (RCT)	Low dose aspirin (LDA) and/or Low Molecular Weight Heparin (LMWH)	Improved pregnancy outcomes (Less PE and IUGR incidence)	[108]
Prospective case-control study	LDA and LMWH in first trimester	Reduced incidence of unexplained recurrent spontaneous abortion	[109]
Database searching for RCTs involving LDA and placebo in PE	LDA or Placebo	LDA reduces PE risk	[110]
Literature searching on LDA and PE	LDA at 100 mg/day <16 gestational weeks	Reduced PE incidence due to LDA prophylaxis	[111]
Systematic literature searches about aspirin and PE	LDA	LDA prophylaxis in at risk patients to develop PE have higher advantages compared to negligible disadvantages i.e., feto-maternal bleeding, aspirin resistance etc.	[112]
Systematic review and an individual participant data meta-analysis	Antiplatelet aspirin therapy in early pregnancy	10–15% reduction in the risk of PE	[113]
A systematic review and meta-analysis of randomized controlled trials	50–150 mg/day aspirin or no treatment at <16 or >16 gestational weeks	LDA at <16 weeks, there was a significant reduction and a dose-response effect for the prevention of preeclampsia	[87]
A systematic review and meta-analysis through electronic database searches (PubMed, Cochrane, Embase).	LDA or placebo at <16 or >16 gestational weeks	<16 weeks, significant reduction of PE. >16 weeks, negligible impact on PE and related disorders	[11]
Databases searching involving keywords ‘aspirin’ and ‘pregnancy’	RCTs that evaluated the prophylactic use of LDA (50–150 mg/day) during pregnancy were included.	LDA initiated at ≤ 16 weeks of gestation is associated with a greater reduction of perinatal death and other adverse perinatal outcomes than when initiated at >16 weeks.	[114]
Meta-analysis of individual patient data recruited to 31 RCTs of PE primary prevention.	One or more antiplatelet agents (e.g., LDA or dipyridamole) versus a placebo or no antiplatelet agent.	Antiplatelet agents were associated with a significant 10% reduction in the relative risk of both PE (*p* = 0.004) and preterm birth before 34 weeks’ gestation (*p* = 0.011) compared to control cases.	[86]
Women at high risk for preterm PE were recruited to RCTs	150 mg/day of aspirin was used to reduce the incidence of aspirin resistance and maximize the effect.	LDA reduced the incidence of preterm PE	[115]
A planned secondary analysis of the Effects of Aspirin in Gestation and Reproduction (EAGeR) trial, a multicenter, block-randomized, double-blind, placebo-controlled trial investigating the effects of LDA on the incidence of live birth.	Daily LDA (81 mg, *n* = 615) or placebo (*n* = 613) and were followed for up to six menstrual cycles or through gestation if they became pregnant.	Preconception LDA appears to be well tolerated by women trying to conceive, women who become pregnant, and by their fetuses and neonates.	[16]
Chronological, cumulative meta-analyses of two recently published meta-analyses of RCTs examining the effects of antioxidant or LDA on the rates of PE.	Antioxidant or Low Dose Acetylsalicylic Acid (LDAA) therapy	Studies with smaller sample sizes are more likely to be biased against the null hypothesis. As such, cumulative meta-analysis is an effective tool in predicting potential bias against the null hypothesis and the need for additional studies.	[116]
Prospective cohort study involving 533 pregnant women in their first trimester	LDAA and LMWH	The use of ASA may be associated with an increased risk of developing a sub-chorionic hematoma (SCH) during the first trimester.	[117]
Multicentre RCTs involving 32 women with a previous delivery <34 weeks gestation with HD and/or SGA and aPLA were included before 12 weeks gestation.	The intervention was daily LMWH with aspirin or aspirin alone.	Combined LMWH and aspirin treatment started before 12 weeks gestation in a subsequent pregnancy did not show reduction of onset of recurrent HD either <34 weeks gestation or irrespective of gestational age, compared with aspirin alone.	[118]
Prospective randomized, placebo-controlled, double-blinded, multinational clinical trial	Daily administration of LDA (81 mg/day) initiated between 6 and 13 weeks of pregnancy and continued upto 36 weeks.	PTB, PE, SGA, perinatal mortality were reduced.	[119]
Prospective RCTs	Preconception LDA daily	It is not associated with reduction of pregnancy loss	[120]
Multicenter, double blind, placebo-controlled trial involving women at high risk for preterm PE	Some of them received 150 mg/day aspirin and some of them received placebo at 11–14 gestational weeks until 36 weeks of gestation	Primary outcome was delivery with PE before 37 weeks of gestation. Treatment with aspirin reduced the incidence of preterm preeclampsia.	[121]

**Table 2:** Updated Research Studies on EVs in PE Pathophysiology.

EVs	Sample Type	Gestational Age	Isolation Method	Pregnancy Condition	Biological Process/Results	Reference
**Maternal Blood Stream and other Body Fluids**						
Trophoblast derived exosomal micro RNA (has-miR-210)	Plasma and HTR-8 cell culture conditioned media	Third trimester	Membrane affinity spin column method	Normal and PE	This micro RNA is responsible for PE pathogenesis	[187]
Exosomes	Plasma	Third trimester (before cesarean section)	Commercial kit (ExoQuick)	Normal and PE	Vascular dysfunction	[194]
Exosomal micro RNAs	Placental mesenchymal stem cells culture conditioned media and peripheral blood	During cesarean section delivery	Ultracentrifugation followed by Real Time PCR	Normal Pregnancy (NP) and PE	High level of exosomal miRNA-136, 494, 495 in PE	[205]
Urinary Exosomal proteins	Urine	After 20 weeks	Centrifugation	Healthy non-pregnant, Normal pregnancy, PE	Phosphorylation of renal tubular sodium transporter proteins that enhance sodium reabsorption in PE compared to NP	[206]
Exosomes	Human umbilical cord mesenchymal stem cells (MSC)	After delivery	Flow cytometry based detection of MSC surface markers	PE	Effect on placental tissue morphology and angiogenesis in rat PE placenta	[207,208]
Placental syncytiotrophoblast derived extracellular vesicles (STBEVs)	Placental perfusate	Following cesarean section delivery	Centrifugation	Normal and PE pregnancy	Lower level of placental protein 13 was found in STBEVs of PE placenta	[195]
Placental extracellular vesicles	Cultured human placental villi explant and Maternal serum	First trimester placenta	Sequential centrifugation and ultracentrifugation	Normal pregnancy	Presence of antiphospholipid antibody increases the level of mitochondrial DNA in the placental EVs and increases the risk to develop PE	[196]
Microparticles	Maternal serum	10–14 weeks	Centrifugation	Normal, PE, IUGR	Serum copeptin, annexin V were higher and placental growth factor was low in PE	[201]
Macovesicles/placental debris	Placental explant and maternal serum	First trimester (8–10 weeks)	Centrifugation	PE	Melatonin is secreted from placental explant that reduce PE sera induced production of endothelial cell activating placental EVs	[209]
Nanovesicles	Placenta	First trimester and term placenta	Differential centrifugation	PE	Transthyretin is increased in amount and incorporated in placental nanovesicles	[210]
EVs	Urine	Maternal urine	EVs were stained for annexin, nephrin and podocin proteins	PE and Normotensive pregnant women	Nephrin protein was packaged in increased amount in urinary EVs of PE women	[211]
Syncytiotrophoblast derived extracellular vesicles (STBEV)	Placental perfusion and maternal plasma	Gestational age matched	Differential centrifugation	Normal and PE	Less nitric oxide synthase in STBEVs of PE women	[202]
EVs	Placental explant	First and second trimester	Sequential centrifugation	Normal and PE	Endothelial dysfunction in severe early onset PE is via soluble angiogenic factors, not by EVs	[212]
Exosomes	Maternal plasma	First, second and third trimester	Differential centrifugation, ultracentrifugation followed by density gradient centrifugation	Normal and PE	The concentration of exosomes is higher and miRNA content is different in PE compared to normal pregnancy	[184]
Microparticles	Placental trophoblasts	At term (>37 weeks)	Two-step centrifugation	Uncomplicated and preeclamptic	Increase MP shedding from PE placenta; upregulation of caveolin-1 and downregulation of eNOS in these MPs which is modulated by vitamin-D	[213]
EVs	Endothelial cells and Platelets	Not mentioned	Differential centrifugation	Normal and PE	Inflammasome activation in placental trophoblasts results in PE development	[204]
**Fetal Circulation**						
Exosomes	Umbilical cord blood	At delivery	Differential Centrifugation + Density gradient centrifugation	Normal	No difference in concentration of exosomes in term, small for gestational age, fetal growth restricted neonates	[198]
Microparticle (MPs)	Umbilical cord blood	At delivery	MPs were identified by size and annexin V fluorescein isothiocyanate (FITC) labelling	Normal and PE	MP levels is higher compared to maternal blood in PE	[199]
Exosomes	Umbilical cord blood	At delivery	Differential centrifugation + Filtration	Normal and PE	Altered protein expression profile that are involved with PE etiology	[200]
